# A phenomenological spiking model for octopus cells in the posterior–ventral cochlear nucleus

**DOI:** 10.1007/s00422-021-00881-x

**Published:** 2021-06-09

**Authors:** Michael Rebhan, Christian Leibold

**Affiliations:** 1 Department Biology II, Ludwig-Maximilians Universität München, 82152 Martinsried, Germany; 2 Department Biology II, Bernstein Center for Computational Neuroscience Munich, Ludwig-Maximilians Universität München, 82152 Martinsried, Germany

**Keywords:** Octopus cells, Natural sounds, Auditory brainstem

## Abstract

Octopus cells in the posteroventral cochlear nucleus exhibit characteristic onset responses to broad band transients but are little investigated in response to more complex sound stimuli. In this paper, we propose a phenomenological, but biophysically motivated, modeling approach that allows to simulate responses of large populations of octopus cells to arbitrary sound pressure waves. The model depends on only few parameters and reproduces basic physiological characteristics like onset firing and phase locking to amplitude modulations. Simulated responses to speech stimuli suggest that octopus cells are particularly sensitive to high-frequency transients in natural sounds and their sustained firing to phonemes provides a population code for sound level.

## Introduction

The auditory brainstem consists of multiple afferent pathways that process different features of sound. Besides the spectral pattern and the temporal fine structure of a sound, it is particularly transients and amplitude modulations (AM), i.e., fluctuations in sound intensity on an intermediate time scale on the order of 10 ms, that provide most information about the identity of a natural sound stimulus [9, 12]. Octopus cells of the posterior-ventral cochlear nucleus (PVCN) are generally thought to encode such amplitude modulations of high-frequency sound stimuli by means of their temporal spike patterns [17, 19]. They are thus likely to play a central role in the processing of natural sounds, including conspecific vocalizations [14, 15, 18].

**Fig. 1:**
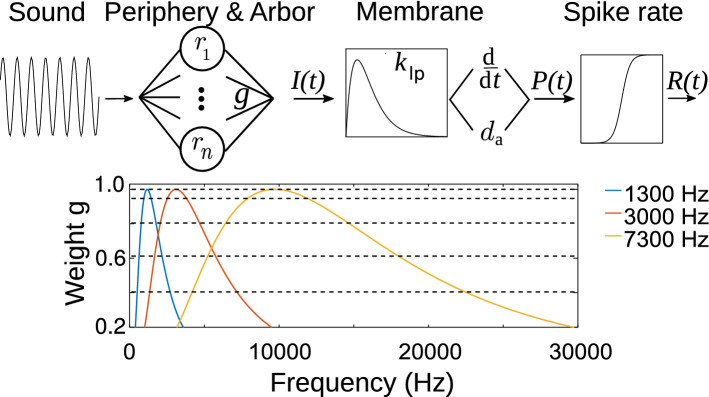
Schematic representation of the octopus cell model. Top: from left to right—a sound pressure wave is used as an input to a modified model of auditory nerve (AN) responses $r_i$ with characteristic cochlear locations $i=1,..,9$. A weighted sum (with weights *g*) of the AN responses is used as an input *I* to the octopus cell that implements a second-order low-pass filter $k_\mathrm{lp}$ and both, a differentiation (d/d*t*) and a proportional $d_a$ component. The resulting potential *P* is transformed to a firing rate *R* using a sigmoidal function. The rate is finally translated to spike times by a Poisson process making *R* a spike probability density over time. Bottom: weight factors *g* for three example octopus model cells with characteristic frequencies (colors) as indicated. The 9 intersections with horizontal dashed lines mark the 9 weights $g_i$ used in the model. Note that, particularly for high characteristic frequencies, the peak of the weight curve is offset to obtain the defined characteristic frequency (color figure online)

Octopus cell spikes only occur at the onset of broad band transients [3–6, 15, 21] but phase-lock persistently to amplitude modulations in a specific AM frequency band. Mechanistically, this firing behavior is thought to arise from integrating across auditory nerve fibers (ANFs) [10, 13, 16] with a broad range of characteristic frequencies [4, 20, 24]. This suggests that the main computation underlying AM extraction is most likely based on the tonotopic pattern of afferent arborization. In addition, octopus cells have remarkably low input resistances of only few Mega Ohms [5, 15] leading to fast enough membrane time constants for processing of fast transient as well as slow amplitude fluctuations. The short membrane time constants are generated by a high density of low-threshold potassium channels [23], which in addition to reducing integration time also endow the neurons with differentiation properties [1, 22] that further facilitate AM locking.

Computational theories of octopus cell function thus require to analyze the interplay between cellular biophysical properties and the circuit parameters describing ANF population inputs. Here, we propose an efficient phenomenological model for octopus cell spiking with only few parameters that are either constrained by direct physiological measurements or functional properties. We find that octopus cell spiking over a wide range of best frequencies can be robustly explained by only small changes in these parameters. Our model thus provides a computationally efficient and robust tool to simulate octopus cell spike responses to any kind of sound stimulus. The model can therefore be used to emulate population inputs to downstream structures in the auditory pathway, the ventral nucleus of the lateral lemniscus and the inferior colliculus.

## Model

The general structure of the proposed effective model is outlined in Fig. 1. In short, the sound stimulus is translated to simulated ANF firing rates $r_i(t)$, where *i* labels the respective frequency channel. The ANF rates are then translated into the octopus cell input by a weighted sum over frequency channels with weight factors $g_i$. The cellular membrane potential is derived from these inputs by a combination of differentiation and low-pass filtering. Finally, the output rate *R*(*t*) of the octopus cell is obtained by a sigmoidal transformation of the pseudo potential *P*(*t*). Spike trains can subsequently be obtained by using *R*(*t*) as the density of an inhomogeneous Poisson process. All individual transformations will be explained in detail in the following paragraphs.

### Arborization

By integrating over ANFs with multiple characteristic frequencies the information about the stimulus’ fine structure is removed, whereas envelope information is preserved. The model generates the cochlear output of an array of ANFs using the (Zilany–Bruce–Carney) model described in [8, 26, 27] with parameters tuned to cat physiology. The model runs with the numerical sampling frequency of 100 kHz. For a model octopus cell with characteristic frequency $f_c$, we simulate 9 frequency channels (corresponding to characteristic cochlear locations) that are logarithmically spaced in the two octave intervals $f_c/2$ to $2\, f_c$. For each ANF frequency, we simulate high, medium and low spontaneous rate fibers and linearly combine them with the fractions, 0.16, 0.24, and 0.6, respectively [11]. The resulting ANF firing probability densities $r_{i}(t)$ are linearly combined to the octopus cell’s input current

1 $$\begin{aligned} I(t)=\sum _{i=1}^9 {g_{i}\, r_{i}(t)}. \end{aligned}$$

The weights $g_i$ are obtained from a log-normal function around the octopus cell’s characteristic frequency (CF, here denoted as $f_{c}$):

2 $$\begin{aligned} g_{i}=\exp \left( -\frac{\left( \log _{2} \frac{f_{i}}{f_{c}+f_{0}}\right) ^{2}}{2\varDelta ^{2}}\right) . \end{aligned}$$

For high frequency cells it is necessary to introduce an additional frequency shift $f_{0}$ to properly fit the observed characteristic frequency, compensating for the overlap of peripheral filters. The parameter $\varDelta$ describes the width (in octaves) of the arborization and will be the essential fit parameter to model the afferent arborization.

Receptive fields of model octopus cells using the plain periphery model either showed unphysiologically strong low-frequency tails [compared to data from [24]], even for cells with high characteristic frequencies, or amplitude modulation locking of the model was distorted by low frequency components from the tails of the ANF receptive fields. For simplicity, we removed these low-frequency components by applying an additional high pass filter

3 $$\begin{aligned} k_\mathrm{hp}(t)=\delta (t) -\frac{\varTheta (t-\varDelta t)}{t}\exp (-2\pi f_\mathrm{hp} t) \end{aligned}$$

to the sound pressure wave before the periphery model, with $f_\mathrm{hp}= 450$ Hz, the Heaviside step function $\varTheta (t)$, and $\varDelta t=1/(100\,\mathrm{kHz})$. This filter suppresses most of the low-frequency-tail of the receptive fields, while still preserving the general response patterns of the model octopus cells (see Discussion for biological feasibility). For numerical convolution, we restricted the kernel $k_\mathrm{hp}$ to a duration of $3/f_\mathrm{hp}$.

### Pseudo potential

The octopus cells respond to rising envelopes of the sound stimuli, which, following [1], we model via a differentiation. The kinetics of the membrane potential response is accounted for by an additional second-order low pass filter

4 $$\begin{aligned} k_\mathrm{lp}(t)=(2\pi \, f_\mathrm{lp}\, t)\,\exp (-{2}\, \pi \, f_\mathrm{lp}\, \varTheta (t))\ \end{aligned}$$

that is supposed to reflect the combination of synaptic and potassium channel kinetics as well as membrane filtering. The frequency $f_\mathrm{lp}$ is the second fit parameter of the model. This leads to the pseudo potential

5 $$\begin{aligned} P(t)=k_\mathrm{lp}(t) *\left( \frac{\text {d}I(t)}{\text {d}t}+2\, \pi \, d_\mathrm{a} I(t)\right) , \end{aligned}$$

which we consider to reflect the behavior of the membrane voltage. The parameter $d_{a}$ allows to include an additional non-derivative component. If $d_a$ is large, the cell has a more primary-like response, whereas for small $d_a$ the response is more onset type. We found that the general dependence on $d_a$ is weak in that for all values $2\pi \, d_{a}\lessapprox 700/\text {s}$ the overall response pattern is onset. Cells with higher CF thereby required larger values to ensure sufficient AM entrainment. For all simulations shown in this paper we satisfy these demands by choosing $2\pi \, d_{a}= \lfloor 50/\mathrm{s} + \frac{f_c-1.3\,\mathrm{kHz}}{10\,\mathrm{Hz}}~\mathrm{1/s}\rfloor _+$.

**Fig. 2:**
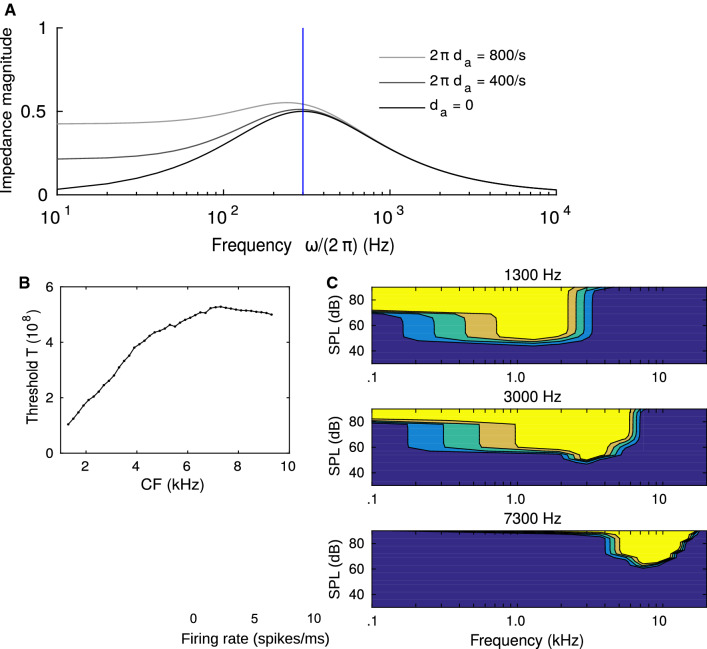
Model design and tuning curves. **a** Impedance magnitude |*Z*| from Eq. (6) displays bandbass characteristics with a peak frequency at $f_\mathrm{lp}$ (blue line for $f_\mathrm{lp}=300$ Hz.). **b** Threshold parameter *T* as a function of CF. The threshold of the activation function $\sigma$ is adjusted to yield $10\%$ spiking probability on a 12 ms pure tone at the assumed pure tone threshold level $L_0$. **c** Tuning curves for three model octopus cells with different best frequencies (as indicated on top). Color-coded are the peak firing rates for pure tones of different frequencies and sound pressure levels. Following [24], here and elsewhere, firing rates are supposed to be interpreted as firing probability densities. Simulations were performed for $f_\mathrm{lp} = 300$ Hz and $\varDelta = 0.9$ (color figure online)

The magnitude of the impedance profile of the pseudo potential *P*,

6 $$\begin{aligned} Z(\omega ) = (\mathrm{i}\omega + 2\pi \, d_\mathrm{a})\, \frac{2\pi \, f_\mathrm{lp}}{(2\pi \, f_\mathrm{lp} + \mathrm{i}\, \omega )^2}\ , \end{aligned}$$

is illustrated in Fig. 2a for the best fitting frequency parameter $f_\mathrm{lp}=300$ Hz (see Fig. 4 below) and illustrates band bass characteristics with peak frequency at 300 Hz (for $d_\mathrm{a}=0$). The time constant $1/(2\pi \, f_\mathrm{lp}) \approx 0.5$ ms fits into the range of membrane time constants in octopus cells [6] and in the auditory pathway in general [2] further supporting the model design.

### Spike generation

To derive the spike rate *R*(*t*) for an inhomogeneous Poisson process from *P*(*t*), we use a sigmoidal function

7 $$\begin{aligned} R(t)=\sigma [P(t)]={\frac{R_{\max }}{\left( 1+Q \exp {\left( -\beta (P(t)-T)\right) }\right) ^{\frac{1}{\gamma }}}} \end{aligned}$$

fixing the rate parameter for the maximal firing rate $R_{\max }$ of the neuron and allowing $Q=(\frac{R_{\max }}{R_{T}})^{\gamma }-1$ to set the firing rate $R_{T}$ at the threshold potential *T*. $R_{\max }=12$ spikes/ms is set to fit the experimentally reported firing probability density when adding a refractory period of 2 ms [24]. Further on, we set $R_{T} = 9$ spikes/ms which yields roughly a 10% probability of an onset spike for pure tone presentation which generates a response *P* equal to *T*.

There is only little data published on the pure tone threshold levels $L_0$ of octopus cells [24], however, these appear to be in a range of 30–60 dB while cells with high characteristic frequencies tend to have higher thresholds. We therefore assumed $L_0$ to grow linearly as

8 $$\begin{aligned} L_0(f_c) = 20\,\left( 2+\frac{f_c-1.3\,\mathrm{kHz}}{6\,\mathrm{kHz}}\right) \, \mathrm{dB\ SPL}. \end{aligned}$$

The threshold parameter *T* is then taken as the peak value of *P*(*t*) for a 12 ms pure tone at CF and threshold level $L_{0}$. The resulting threshold parameters are shown in Fig. 2b.

The slope parameters $\beta$ and $\gamma$ of the sigmoid take values of $2.5/(6\pi )\cdot 10^{-6}$ and 1/2, respectively. This parameter choice generates relatively steep activation functions, which ensure suppression of spontaneous and sustained firing owing to the non-derivative component from Eq. (5). Furthermore, since in model cells with low CF the pseudo potential *P*(*t*) fluctuates in only a small interval, the steep slope also ensures that the small dynamic range of *P* is translated to the full output range of *R*.

The parameters of the sigmoid not only ensure that the model responds reliably and temporally precise to pure-tone stimuli above threshold. The specific choice of the parameters $\beta$ and *T* was also checked to not introduce unphysiological islands in the receptive fields.

### Basic response properties

**Fig. 3:**
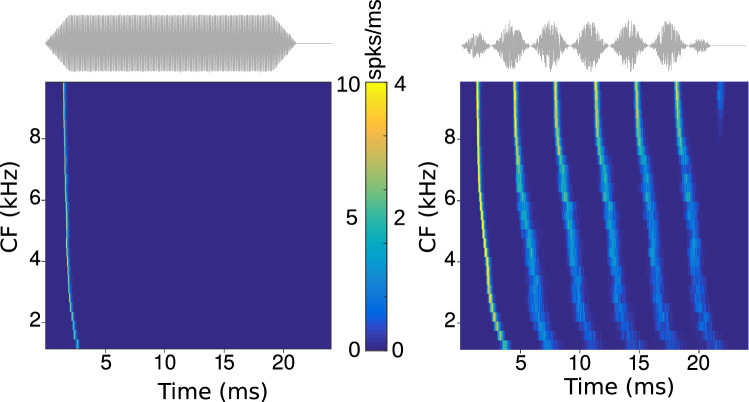
Firing rates (color-coded) of the modeled octopus cells with different characteristic frequencies CF for two representative stimuli (top: gray). Left: pure tone stimuli of 80 dB SPL with their frequencies matching the characteristic frequency of the octopus cell. Right: sinusoidal modulated white noise stimulus of 20 dB above threshold and a modulation frequency of $f_{m}=300$ Hz and a modulation depth of $100\%$. Top panels show example sounds (gray) to illustrate the applied stimulus envelopes. Simulations were performed for $f_\mathrm{lp} = 300$ Hz and $\varDelta = 0.9$. Noise level of 20 dB above threshold roughly corresponds to 80 dB SPL and was chosen to match the levels used in physiological recordings in [17]. Color scales are normalized to the maximum (left: 10/ms, right: 4/ms) (color figure online)

During early electrophysiological experiments as well as while designing our model, pure tone stimuli are a necessary simplification to probe octopus cell responses in a controlled way (also as compared to AM stimuli) although these stimuli only provide sketches of these cells’ function under realistic acoustic conditions. Nevertheless, pure tones provide a valuable good first benchmark for modelling. We thus confirmed that the model’s responses can fit octopus cells’ localized pure tone receptive fields (Fig. 2c), onset responses to pure tones (Fig. 3 left), and locking to sinusoidally amplitude modulated sounds stimulus (Fig. 3 right). As by design, the model only fires at the onset of the pure tone, whereas it phase-locks to each cycle of the amplitude modulated sound.

## Mathematical analysis of a simplified model

To better understand the model dynamics, we analyze its linear part *P*(*t*) for an amplitude modulated pure tone stimulus

9 $$\begin{aligned} s(t) = \varTheta (t-t_{0})\, A(t)\, \sin (2\pi f_s t). \end{aligned}$$

Here, $\varTheta$ represents the Heaviside function that implements the onset at time $t=t_0$. The carrier frequency is denoted by $f_s$. The amplitude *A*(*t*) is assumed to vary much more slowly than the carrier and thus can be taken as constant $A(t) \approx A$ during the period $1/f_s$.

Due to its combination of low pass and band pass filtering the response of a single ANF to a pure tone stimulus (with frequency $f_s$) can be approximated as

$$\begin{aligned} r_{i}(t) \approx A(t)\, [a_{i}(f_{i},f_s)+b_{i}(f_{i},f_s) \sin (2\pi f_s\, t +\phi _i)], \end{aligned}$$

with a constant component $a_{i}$ and the oscillatory component proportional to $b_{i}$. With this, Eq. (1) for an octopus cell with CF $f_c$ can be rewritten as

10 $$\begin{aligned} I(t) =\varTheta (t-t_{0}) A(t)\, [a_{g}(f_{c},f_s)+b_{g}(f_{c},f_s)\, \sin (2\pi f_s t + \phi _{c,s})]\nonumber \\ \end{aligned}$$

with

11 $$\begin{aligned} a_{g}(f_{c},f_s)=\sum _{i}{g_{i}(f_{c}, f_{i})a_{i}(f_{i},f_s)} \end{aligned}$$

and

12 $$\begin{aligned} b_{g}(f_{c},f_s)=\sum _{i}{g_{i}(f_{c}, f_{i})b_{i}(f_{i},f_s)} \end{aligned}$$

**Fig. 4:**
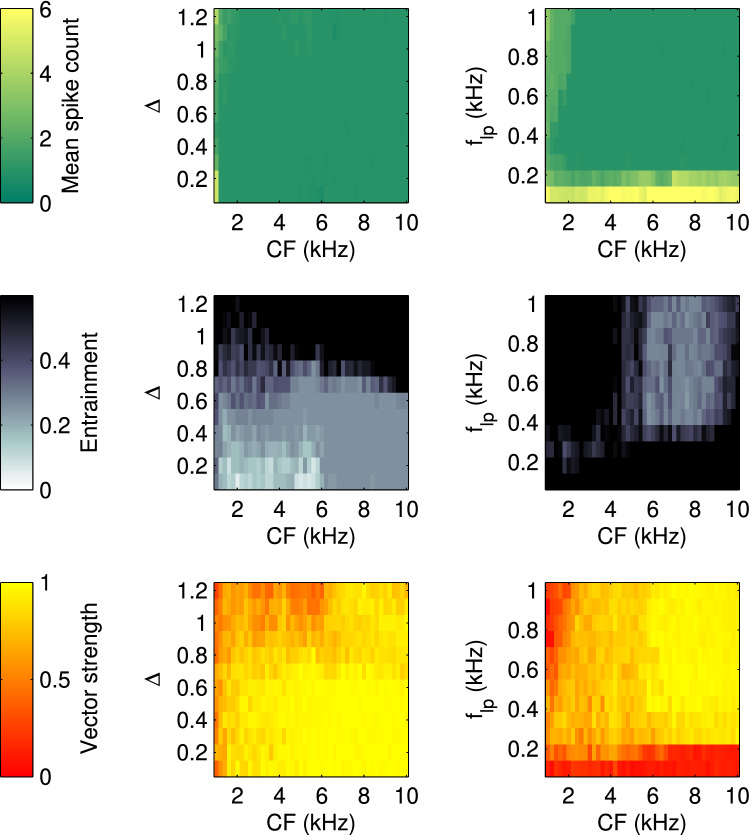
Effects of arborization width $\varDelta$ (left panels) and low pass frequency $f_\mathrm{lp}$ (right panels) and as a function of characteristic frequency (CF). Top: the average number of spikes as response to pure tones (at best frequency). Middle: entrainment to a 300 Hz sinusoidally amplitude modulated noise (as in Fig. 3). Bottom: vector strength of the response to the same stimulus as in the middle

**Fig. 5:**
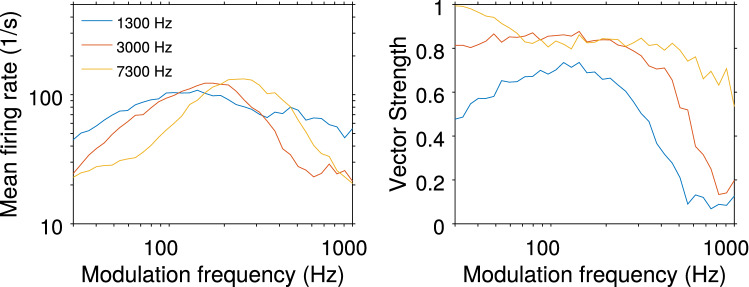
Responses of octopus cells with different characteristic frequencies (as indicated) to sinusoidally amplitude modulated noise ($100\%$ modulation depth, 20 dB above threshold, duration: 25 ms or at least 10 modulation cycles). The modulation frequency is shown on the *x*-axis. The left panel depicts the mean firing rate in response to the stimulus. The corresponding vector strength is shown on the right

**Fig. 6:**
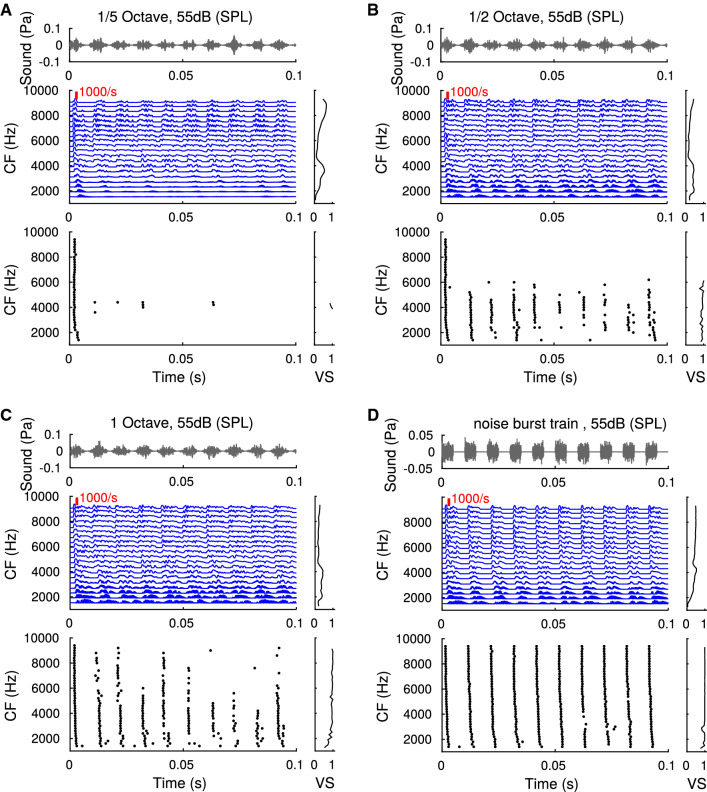
Amplitude modulated band-pass noise and a train of white noise bursts. Responses of ANFs (blue) and octopus cells (spike rasters) with different characteristic frequencies (as indicated) to sinusoidally amplitude modulated bandpass noise (fourth order gamma tone filter with center frequency 5 kHz) and a train of noise bursts (duty cycle 5 ms, cosine ramp 100 $\upmu$s). Modulation/burst frequency was 100 Hz in all panels. Graphs on the right depict vector strength as a function of characteristic frequency (color figure online)

**Fig. 7:**
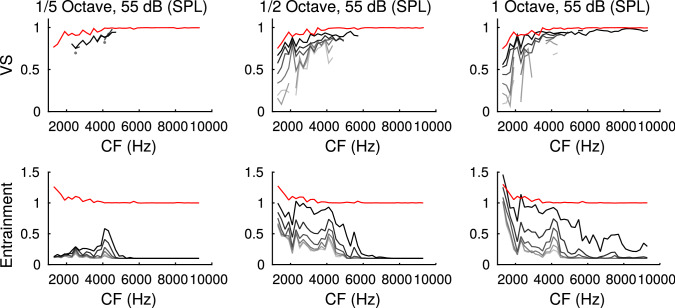
Sensitivity to amplitude modulations. Three versions (columns) of sinusoidally amplitude modulated bandpass noise with different bandwidth (same as Fig. 6) are evaluated for their capacity of temporally faithful (top: vector strength) and secure (bottom: entrainment) amplitude modulation detection. Grey levels depict varying percentages of modulation depth (gray to black: 0, 20, 40, 60, 80, 100). Red lines indicate values obtained from noise-burst stimulation (see Fig. 6). All plots are averages from 50 repetitions of the stimuli (color figure online)

**Fig. 8:**
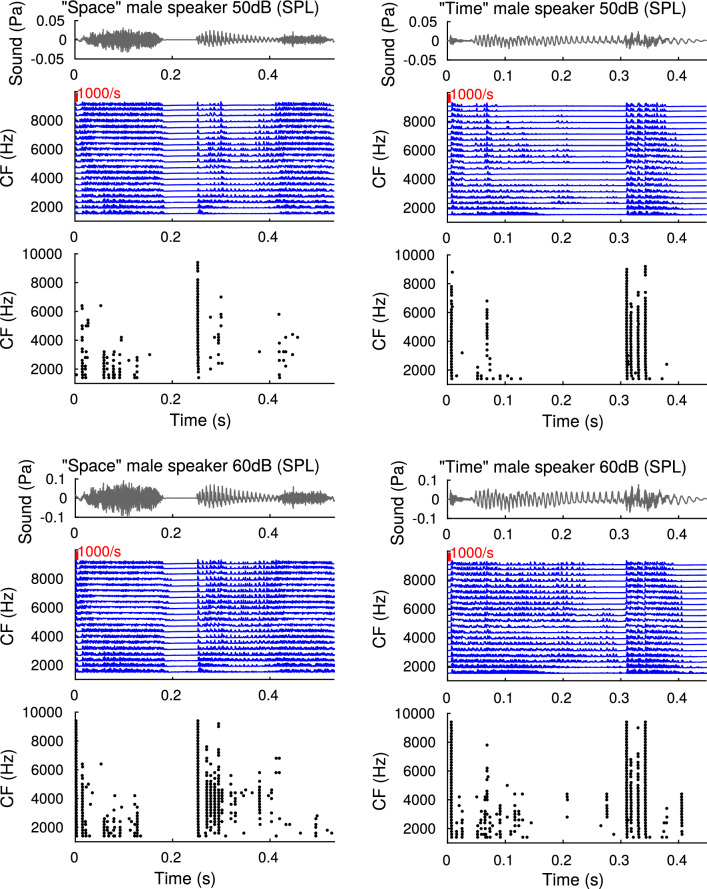
Responses to speech sounds. Panels are outlined as in Fig. 6 with ANF firing probability densities (blue) and octopus spike raster plots (black). As stimuli, we applied two speech signals (“Space” and “time”) at 50 dB SPL (top) 60 dB SPL (bottom) (color figure online)

Due to the filtering properties of the periphery model $a_{i}$ grows with $f_{i}$ while $b_{i}$ decays. Since octopus cells seem to be most consistently modeled with input mostly from high frequency ANFs [25], one can neglect the $b_{g}$-component of Eq. (10) and simplify it to:

13 $$\begin{aligned} I(t) \approx \varTheta (t-t_{0})\, A(t)\,a_{g}(f_{c},f_s) \end{aligned}$$

The pseudo potential *P* is mostly governed by the derivative component of Eq. (5) [1]. Thus for simplicity, in the next paragraph we will assume that $d_{a} \approx 0$.

Using the input approximation (13) for a non-modulated pure tone ($A(t)=A=\mathrm{const.}$), Eq. (5) simplifies to a delta-like pure tone (PT) response

14 $$\begin{aligned} P_{\text {PT}}(t) \approx A\, k_\mathrm{lp} *\delta (t-t_{0})\, a_{g}(f_c,f_s){.} \end{aligned}$$

Conversely, for an amplitude modulated stimulus, the differentiation of the input approximation (13) by Eq. (5) yields an additional additive component extracting the derivative of *A*(*t*),

15 $$\begin{aligned} P_{\text {SAM}}(t)=P_{\text {PT}}(t)+ k_\mathrm{lp} *\varTheta (t-t_{0})a_{g}(f_c,f_s) \frac{\text {d}}{\text {d}t} A(t).\nonumber \\ \end{aligned}$$

The term $\propto \frac{\text {d}}{\text {d}t} A(t)$ lets the octopus neurons fire on the positive slope of their inputs. This behavior enhances fast amplitude modulations, particularly at high modulation frequencies (as concluded from a previous octopus cell model in [7]). It thus accounts for phase locking to sinusoidally amplitude modulated tones up to a certain modulation frequency (governed by the second-order low-pass kernel $k_\mathrm{lp}$). Therefore, by design, the model can replicate the essential firing characteristics for pure tone and AM stimuli.

What remains open is, how to find the parameters that reliably produce such physiological responses. Since $R_\mathrm{max}, Q, \beta , T, d_a, \gamma$ are constrained by basic cellular measurements (see above) we next will explore the effect of the two thus far unconstrained parameters $f_\mathrm{lp}$ and $\varDelta$ by simulations.

## Simulations

The above considerations leave only two parameters that are not directly constrained by the desired firing pattern.

1. The width of the arborization $\varDelta$ (Eq. 1) and
2. the cut-off frequency $f_\mathrm{lp}$ of the low pass filter Eq. (4).

Both were scanned by simulations shown in Fig. 4 monitoring the quality of three functional criteria for a given parameter configuration.

The first criterion (upper panels) is the mean number of spikes for an 80 dB SPL pure tone stimulus at the octopus cell’s best frequency. A mean spike count of 1 indicates that the cell produces the experimentally reported onset response. A mean spike count of about one is realized across CF by all $\varDelta$-values between 0.4 and 1 and $f_\mathrm{lp}>250$ Hz. The exception are cells with very low CF, which exhibit low levels of sustained firing in general.

A second criterion is the entrainment *E*, which is defined as the fraction of spikes per modulation cycle of a sinusoidal amplitude-modulated noise stimulus (in this case with a modulation frequency of $f_{m}=300$ Hz). High values of *E* are thus desirable. We find generally low values of *E* at $\varDelta <0.8$ and at $f_\mathrm{lp}>350$ Hz, which excludes these parameter regimes from further considerations.

As a last criterion we use the vector strength $V=\frac{1}{n}|\sum _{j=1}^{n}{\exp ({2\, \pi i f_{m} t_{j}}}|)$, where $t_{j}$ is the time of the *j*-th spike and *n* is the total number of spikes. It indicates the cell’s ability to phase-lock to amplitude modulations. High values of *V* indicate good phase locking and are preferable for choosing parameters. Low values of *V* are generally only observable for $\varDelta <1.0$ in the mid-CF range, and for $f_\mathrm{lp}<100$ Hz, which would also exclude these parameter regimes.

As a result of these considerations we suggest as a good choice for the model parameters $f_\mathrm{lp}=300$ Hz and $\varDelta = 0.9$, where none of the criteria exhibit extensive regions of low unfavorable values along the CF axis.

### Modulation transfer functions

Phase-locking of the model responses to varying AM frequencies are experimentally quantified by modulation transfer functions [17]. We thus also applied AM stimuli with different carrier frequencies to our model (Fig. 5) reproducing experimentally reported results.

Cells show a typical best modulation frequency with maximal firing rate [17] and the best modulation frequency correlates with the characteristic frequency in the tested range as expected from peripheral filtering. All cells phase lock very well to the AM stimuli over a broad frequency range as indicated by vector strength. Above some threshold frequency cells cease to phase lock, and this threshold frequency again correlates with characteristic frequency for the range tested.

### Transients

Octopus cells have been proposed to particularly encode sound transients [5, 15] that occur on a much faster time scale than the amplitude modulations in the stimuli tested so far. The transition from amplitude modulations to transients can be explored by manipulating spectral width of the carrier noise and modulation depth. We first simulated model responses to amplitude modulated noise with increasing bandwidth and full modulation depth and compared them to the responses to a sharp-onset noise-burst stimulation (Fig. 6). For fixed sound level and increasing spectral width, octopus cells gain in response rate (entrainment) and vector strength, surpassing ANF vector strengths. This finding corroborates that octopus cells are best driven by sharp broad band transients.

To also quantify how well octopus cells are able to detect amplitude modulations, we next varied modulation depths (Fig. 7). Phase locking (if present over all CFs) only started to clearly deviate from control levels (zero modulation depth) at at least 60% modulation depth and almost achieved vector strengths obtained with noise-burst stimulations. Conversely, entrainment required at least about 80% to show marked differences to controls but, particularly for high frequencies, remained below entrainment obtained from noise burst responses.

We thus conclude that amplitude modulations generally yield good phase-locking of octopus cells, whereas they do not reach the efficiency of sharp transients in evoking secure responses, particularly for narrow band stimuli and at characteristic frequencies above 5 kHz.

### Complex stimuli

To compare our results with bandpass noise to natural stimuli, we also applied it to two exemplary speech sounds. Figure 8 shows the results for the speech stimuli “‘time”’ and “‘space”’ from a male speaker, presented at 50 dB and 60 dB SPL.

In line with our observations from bandpass noise, the qualitative observation from these plots is that the octopus cells fire selectively on sharp transients, the strong modulations as well as parts of the stimulus with a broad spectrum, as typical for consonants. Cells with different characteristic frequencies thereby select different parts of the stimuli: The pattern is bipartite, in that cells with characteristic frequency above some threshold only show responses to sharp transients. This frequency threshold is raised by increasing stimulus level, and also reflects the recruitment of high frequency ANFs. As a result, the threshold of sustained octopus cell firing to phonemes approximately coincides with the CF at which the stimulus intensity matches the cells’ pure tone threshold according to Eq. (8).

## Discussion

Owing to the high technical challenges in both in-vivo and in-vitro physiology preparations, octopus cells, despite their putative crucial role in the processing of natural sounds [15, 18], have not been investigated to a similarly large extent as other cells in the ascending auditory brainstem, like, e.g., bushy cells, or principal cells of the MNTB and SOC. We therefore still have a very incomplete picture on how the octopus cell pathway processes sound. Here, we present a modeling approach complementing detailed physiological studies comprising a phenomenological computational model for a population of octopus cells. The model is constrained by the well-studied responses of octopus cells to pure tones and amplitude-modulated noise and implements their basic known physiological operation of a differentiation of its input. Such a phenomenological approach allows us to test functional hypotheses on large sets of natural stimuli and thereby generate new hypotheses for follow-up experimental studies.

The disadvantage of a phenomenological approach is, however, that making connections to the underlying biological substrate is not always straightforward. For example, we do not have a good justification for requiring an additional high-pass filter applied before the established periphery model [8, 26, 27] other than improving of how the model fits the frequency tuning curve and phase locking data. A possible explanation may be additional mechanisms that suppress low frequency inputs to octopus cells. Despite this drawback, our model makes a clear experimentally-testable prediction, viz., the neurons should cease sustained firing to ongoing amplitude modulations of a complex stimulus (such as those evoked by phonemes) if the stimulus intensity (integrated over the whole word) falls roughly below the cell’s threshold to pure tone stimulation at CF, whereas they should only fire to sharp transients if the stimulus intensity is above the cell’s CF pure tone threshold (Fig. 8).

So far, the pathway originating from octopus cells was hard to probe in a functional manner, since the link between acoustic stimulus and synaptic input was unclear. A major benefit of our model is that it allows us to generate physiologically realistic inputs for physiological studies of such downstream neuronal structures, e.g., the ventral nucleus of the lateral lemniscus or the inferior colliculus.

Our simulations so far have already shown that the octopus model responds strongly and selectively to rapid signal onsets in speech stimuli (Figs. 6, 8). This suggests that speech stimuli, with abundant sharp transients and strong modulations, seem to be a suitable test set for eliciting rich activity for octopus cell recordings. Moreover, ethologically relevant sounds, such as conspecific vocalization, rustling, and predator noise all contain transients and broad band contributions and thus octopus cell populations are likely to play a crucial role for audition in natural environments in general.

## Declaration

### Code availability

The paper is based on custom code that is made available upon reasonable request to the corresponding author.
